# Vulnerability, equity and universal coverage – a concept note

**DOI:** 10.1186/1471-2458-12-S1-S2

**Published:** 2012-06-22

**Authors:** Pascale Allotey, Sharuna Verghis, Fatima Alvarez-Castillo, Daniel D Reidpath

**Affiliations:** 1 School of Medicine and Health Sciences, Monash University, Sunway Campus Malaysia; 2 College of Arts & Sciences, University of the Philippines, Manila, The Philippines

## 

Universal coverage embodies a critical underlying social value; the recognition that those goods and services that support health, however we define it, have to be available and accessible to all. The choice of which of these goods and services are considered essential, the mechanisms by which they would be financed and so on are almost secondary to this basic consensus, that coverage has to be universal, leaving no one out. Inextricably linked to this understanding of universal coverage is therefore an imperative to ensure equity.

Within the context of health and health systems, pursuing equity requires the identification and addressing of those determinants that systematically restrict or prevent access to particular groups. Pursing equity also requires the institution of processes that enable population groups to help to identify the systems that perpetuate inequities as well as possible solutions to reduce or eliminate disparities.

The notion of vulnerability, although extensively used (and sometimes abused) in the public health and social sciences literature [1,2], provides a useful framework for identifying various population groups and systems against which achievement of universal coverage can be benchmarked. In this background paper, we attempt to bring to the fore, some of the issues that create vulnerability and that define the populations critical to universal coverage. We argue that contemporary discussions on universal coverage are often overwhelmed by considerations of financing to the detriment of the central tenet of what universal coverage tries to achieve: health for all.

## Vulnerability

In public health and in relation to health care, vulnerability is broadly described as the inability to substantially protect oneself from potential harm [3], ‘the susceptibility to harm’ resulting from the interaction of risk factors and supports and resources available to individuals and groups [4] (p 1220), and the ‘progressive loss of wellbeing, i.e. health’ related to social and economic deprivation [5].

As such, vulnerability is often contextual, dependent on social and cultural systems and political and economic trends. Groups experience social vulnerability based on shared racial, ethnic, cultural, and situational similarities. The paper by Ravindran in this series for instance, provides a critical analysis of the importance of gender in defining and assessing effects of vulnerability and of equity in achieving universal coverage. The capacity to cope with risks that endanger health is further compromised by poor access of the group to resources and information; social support and social networks; political participation and representation; and access to social safety nets [6,7]. Social vulnerability is also created by discrimination, isolation, and human rights violations [8]. The homeless, elderly, refugees and migrants, sex workers, people with mental illnesses, disabilities and chronic illnesses, sexual minorities, and the medically uninsured are some groups that experience social vulnerability because of the common resource and capacity deficits brought about by their shared racial, ethnic, cultural and geographical similarity. Social vulnerability may also play out through shared physical spaces such as neighbourhoods with high violence and crime rates or conflict affected areas. Groups that are vulnerable because of deficits in their collective access to resources also tend to experience poorer health outcomes [8-10].

An exploration of migration laws, both at the national and international levels, provides an important example of how growing sub-populations in many countries are often outside considerations of universal coverage. A multi-country study of Asian women migrant workers [11] found that their vulnerability to HIV was related to policies that ban outmigration of women and promote irregular migration; gender selectivity in migration policies that create and sustain a gendered international division of labour with women in informal and unprotected work sectors; poor access of migrant women to credit leading to economic dispossession and indebtedness in financing out-migration; poor regulation of recruiting agents and other intermediaries; labour rights violations within unrecognized and unprotected work sectors; immigration policies in destination countries that make pregnancy, STIs, tuberculosis and HIV terms for deportation; and national HIV and AIDS programs that exclude migrant workers.

## Vulnerability in health systems

The World Health Organization (WHO) refers to health systems as “all the organizations, institutions and resources that are devoted to producing health actions” [12]. It includes patients, their families and communities, the Ministry of Health, health financing bodies, behaviour change and vector-control programmes, health services organizations, pharmaceutical companies and others. It also includes intersectoral actions across government that impact on health [13]. The goals of health systems include “improving health and health equity in ways that are responsive, financially fair, and make the best, or most efficient, use of available resources” [14].

As entities, health systems may also be vulnerable – at a macro level vulnerability occurs when they are marginalised within political and national priorities; and at a macro level where the pillars that make up the health system are fragile. Robust health systems moderate the effects of health risks and vulnerabilities [15] by providing a continuum of health services including primary, secondary and tertiary prevention, as well as other intersectoral actions to protect and promote health. Care is not guaranteed simply by the existence of medical facilities [12] (p 9), the facilities in turn need to be strong and equitable to cope with potential access by all.

Vulnerability of health systems translates into reduced protective resources and poor health outcomes for individuals and groups, and forfeiture of potential national socio-economic gains. In addition, the population level and global dimensions of health problems witnessed in relation to HIV and AIDS, severe acute respiratory syndrome (SARS), and recent disasters as in Haiti and Fukushima are also a reminder of the significance of the resilience and vulnerability of health systems in relation to population and global health.

Governance is another significant factor related to the vulnerability of health systems and requires increasing government effectiveness; control of corruption; and participation from all stakeholders including users [16]. Corrupt practices occur across a continuum ranging from inefficiency and minor mismanagement to absenteeism of health workers from work, ‘under-the table’ payments for public services and procurement of supplies, and the absence of regulatory policies, standards and mechanisms [16]. Government effectiveness includes efficiency and adequacy of capacity of technical and administrative skills of public institutions and servants [17], competent discharge of its roles, coherent internal policy and program formulation, and consistent external coordination with other sectors. The World Health Report 2010 states that about 20-40% of health spending is wasted which could otherwise be used to achieving universal coverage [18]. Voice to the people is an important form of external accountability and ensures participation and a collaborative model of governance. However, it depends on the democratic freedoms accorded by the political system, a national anti-corruption strategy, robust regulatory frameworks and enforcement by the government, and the independence of the judiciary and the media among other things. Accountability helps to keep in check corruption, inefficiencies and ineffectiveness.

## Conclusion

This paper discussed the vulnerability of people and health systems. For policies that aim to achieve universal coverage, an understanding of vulnerabilities facilitates the casting of a very wide net to cover as many as can be identified who may be in situations not well characterised by mainstream services. Vulnerability draws attention to political, social, legal and cultural contexts that spawn exclusions and deprivation, and communal and inter-personal spaces that encourage subordination, neglect and discrimination.

Similarly, an assessment of the vulnerabilities of health systems provides a useful tool to assess the ability of the health system to withstand the pressures of various population groups. The vulnerability of health systems underscores the importance of good governance, balanced and equitable development, and peace in achieving positive health outcomes and building robust health systems. Risks to the resilience and integrity of people and health systems reflect structural inequalities and inequities which need to be addressed strategically and with urgency.

### Competing interests

The authors declare that they have no competing interests.
